# Super-Resolution Reconstruction of High-Resolution Satellite ZY-3 TLC Images

**DOI:** 10.3390/s17051062

**Published:** 2017-05-07

**Authors:** Lin Li, Wei Wang, Heng Luo, Shen Ying

**Affiliations:** 1 School of Resource and Environmental Sciences, Wuhan University, Wuhan 430079, China; lilin@whu.edu.cn (L.L.); wangwei_sres@sina.com (W.W.); 2 Geomatics Center of Guangxi, Guangxi Bureau of Surveying, Mapping and GeoInformation, Nanning 530023, China; luoheng330@hotmail.com

**Keywords:** super-resolution, image reconstruction, three-line camera satellite, ZY-3, remote sensing

## Abstract

Super-resolution (SR) image reconstruction is a technique used to recover a high-resolution image using the cumulative information provided by several low-resolution images. With the help of SR techniques, satellite remotely sensed images can be combined to achieve a higher-resolution image, which is especially useful for a two- or three-line camera satellite, e.g., the ZY-3 high-resolution Three Line Camera (TLC) satellite. In this paper, we introduce the application of the SR reconstruction method, including motion estimation and the robust super-resolution technique, to ZY-3 TLC images. The results show that SR reconstruction can significantly improve both the resolution and image quality of ZY-3 TLC images.

## 1. Introduction

Tsai and Huang [1] introduced the idea of super-resolution (SR) in 1984 for the multi-frame image restoration of bandlimited signals. Research on SR is part of the image and signal processing study field, which includes two different branches. One branch aims to improve the interpretability of an optical imaging system, and the other focuses on reconstruction to obtain a higher-resolution image from low-resolution images [2]. Informative overviews of existing algorithms are provided by Borman and Stevenson [3] and Park et al. [4].

From the remote sensing aspect, most studies have involved the latter branch, i.e., remote sensing image SR reconstruction [3,4,5]. In the past 20 years, the SR technique used in the remote sensing area has quickly developed. Classical algorithms and models have been proposed by different researchers. Two kinds of SR processing methods can be distinguished: single-frame and multi-frame. In the single-frame method, only a single image of the objective scene is used to obtain the super-resolved output. In the multi-frame method, several low resolution images of the same scene acquired at different condition are used to create a higher resolution result [6]. Multi-frame SR methods have been widely applied in high-resolution remote sensing, without or in combination with the multi-angular acquisition capability some of these sensors have [7,8].

Some applications have taken advantage of SR techniques in practical use to improve image resolution. In our study, the image resolution refers to the spatial resolution. For example, the SPOT5 satellite uses its “Super-mode” to achieve 2.5 m to 3 m resolution images from its 5 m resolution camera [8]. This satellite uses a camera design incorporating SR, serving as an example of a hardware application of SR. As with the SPOT5 satellite camera, some techniques involve high costs and complex technology; thus, these techniques are very difficult to implement for most applications. As a result, SR techniques based on algorithms and processing models are more useful and practical for most image processing conditions.

In many applications today, image resolution has become very important. While being a strong marketing argument for entry-level digital cameras, a high-resolution sensor can greatly improve image quality if combined with good optics on a high-end digital SLR (Single Lens Reflex) camera. Moreover, higher resolution in surveillance cameras can make it possible to see much more detail, which could, for instance, facilitate an investigation. Unfortunately, increasing the resolution at the sensor level requires the purchase of new equipment, which might be financially prohibitive. With the goal of improving the resolution for satellite image, more images acquired from similar condition is considered to be used.

Low resolution limits the usage of remotely sensed image, meanwhile satellite image with higher resolution can provide greater detailed information for different types of application. A method of using similar images to achieve higher resolution is needed. For example, reconstructing a higher resolution image with some image of the same scene, and aggregating more information. The ideal scenario is one in which we can obtain four images, with the last three being shifted from the first image by 0.5 pixel on the *x*-axis, 0.5 pixel on the *y*-axis.

There have been some studies using SR method in different application field, such as forestry, agriculture, land cover detection and so on [9,10,11]. For example, SR method was used in estimating the missing high frequency details from infected leaf image and helped farmers detect exact leaf diseases [9]. Study in [10] investigated the adequacy of a remote sensing instrument spatial resolution in monitoring crop growth in agricultural landscapes with different spatial patterns. Ling et al. [11] introduced a SR mapping method to produce a fine-spatial-resolution land cover map from coarse-spatial resolution remotely sensed imagery.

For the ZY-3 Three Line Camera (TLC), which provides three images from slightly different angles almost simultaneously, SR reconstruction can provide a higher-resolution image. The ZY-3 TLC SR image is intended to significantly improve the resolution of standard ZY-3 panchromatic images. This study attempts to implement SR processing for ZY-3 TLC images, and the efficacy of the technique is evaluated.

There have been some studies on the SR technique for satellite images using a processing algorithm. Borman and Stevenson [3] and Park et al. [4] provided informative overviews of existing algorithms for SR. SR algorithm includes two main steps, registration and reconstruction. Registration is about the alignment of set of images within the same spatial reference, and the reconstruction is establishing a new image with higher resolution from the aligned images.

About the image registration method, Zitová and Flusser [12] provide a review for related literatures. Camera motion and change of focus are two important factors existed in the difference between the low-resolution input images. These two factors should be considered accurately. Without accurate registration, the final reconstructed image may be blurred, because the original signals are not correctly represented pixel by pixel. Thus SR algorithm requires subpixel image registration, which could promise good effect for image reconstruction. Most of the studies complete image registration via spatial method or frequency domain. The Fourier transform is a well-known frequency-domain method, which is limited to global motion models. Planar shifts, rotation, and scale are often expressed in the Fourier domain. These factors are normally considered in the algorithm models. Moreover, compared with spatial domain, aliasing is also an important factor that can be handled in the frequency domain, and it is easy to describe.

Multiple, possibly transparent or occluding motions in the processing set of images are considered in the study of Irani [13]. With the help of planar motion, this study makes an attempt to use the iterative multiresolution approach for estimating the image motion. In the study of Gluckman [14], the method of computing planar rotation from gradient field distribution of the images is described, which facilitates the registration of image. In all, with fewer parameters and simpler calculation, the planar model is more robust in the presence of noise during the processing. Some studies considered planar rotation as part of camera motion, and extended the planar shift motion model to include planar rotations [15,16,17,18]. Though very small, these rotations affect the result of final registration.

In this study, to achieve a rapid and accurate image, a frequency domain technique based on low frequency and aliasing-free components [16] is used for the motion estimation of ZY-3 TLC images. We use the property that a shift in the space domain is translated into a linear shift in the phase of an image's Fourier transform to register the ZY-3 TLC images, which are slightly different in their acquisition time and angle.

Spatial domain image reconstruction includes projection onto a convex set (POCS), iterative back projection (IBP), and structure-adaptive normalized convolution. These methods are usually very sensitive to their assumed model of data and noise, which limits their utility [17]. Many different iterative methods have been proposed in the past two decades (e.g., [19,20,21,22,23,24]). Zhang and Huang [20] found out that all the component substitution and multi-resolution analysis methods based on multi-images can be derived from the Bayesian fusion and demonstrated them with QuickBird and WorldView-2 images. According to Elad and Hel-Or [22], non-iterative methods have been addressed, assuming that correct estimations of motion and blur are available. These methods have a wide range of complexity, memory and time requirements. None of the above methods address noise models other than Gaussian additive noise, and regularization is either not implemented or is limited to Tikhonov regularization. Considering outliers, Zomet et al. [24] have proposed a successful, more robust method without proper mathematical justification. The difference resides in the computation of the gradient, which is not given by the sum of all errors but, rather, by the median of all errors. This calculation provides robustness against outliers in the low resolution images.

Therefore, to reconstruct the low-resolution registered ZY-3 TLC images rapidly and robustly, we use the robust SR technique proposed by Zomet [24].

## 2. Data and Methods

### 2.1. Data

ZY-3 high resolution land resource observation satellite is launched on 9 January 2012. With the purpose of implementing earth observation and stereo mapping, the ZY-3 satellite is equipped with a 2.1-m panchromatic nadir camera, and 3.6-m forward and backward cameras. These cameras all have a wavelength of 450 nm–800 nm, according to [25]. The three cameras equipped in ZY-3 satellite platform were combined together to make a three line array camera (TLC). TLC images are useful in generating a stereo image and can be further processed to create a digital elevation model (DEM) [26].

In this study, clips of ZY-3 TLC images that cover a 7.44 × 5.15 km^2^ area (3543-pixel by 2453-pixel area for nadir image) are used for the SR experiment. The resolution of the nadir image is 2.1 m, while that of the backward and forward images is 3.6 m. All images are of good quality with no cloud coverage, no bad line, and no blurring. The images used in our study are acquired from the plain area on the central part of China. Imagery of a plain area has higher geometric accuracy compared with that of a mountainous area. This imagery has both agricultural areas and residential areas, which are suitable to represent the image quality and characteristics, and are useful in comparing the effects of the original image and processed image. With this set of images, we tested the results of SR processing with the goal of achieving a new image with high resolution and better interpretability.

### 2.2. Methods

We first ortho-rectified the ZY-3 TLC images using the RPC (rational polynomial coefficients) file. Then, to ensure the same resolution for the low-resolution TLC images, we resampled the 3.6 m forward and backward images to a 2.1 m resolution nadir image. In this way, the recording of the three TLC images could be completed correctly and the details of the higher-resolution nadir image were retained. Then, rotation estimation and shift estimation are calculated as two motion estimation steps that make the resampled image align with the nadir image. Based on the results of motion estimation, robust SR method was used for achieving a high resolution image.

#### 2.2.1. Motion Estimation

For motion estimation, alignment of the similar input images accurately is very important, and is considered to be the essential aspect of the related algorithms. To achieve a good performance of the image registration, the low resolution images should be correctly sampled, and should not have aliasing artifacts. In the study of Vandewalle [15,16], to precisely register the input set of aliased images, a frequency domain technique based on low frequency and aliasing-free components is proposed.

In the phase of Fourier transform of an image, it is possible to use the property that a shift in the space domain is translated into a linear shift. Also, the space domain rotation can be visible in the amplitude of the Fourier transform. In the study of [15,16], computations of Fourier transforms of images are made, and then in both amplitudes and phases the 1-D shifts are determined. In terms of results, this method is more robust, because it discards the high frequency components, where aliasing may occur.

We used a frequency-domain algorithm to estimate the motion parameters between the reference image and each of the other images. Only planar motion parallel to the image plane was allowed.

The main steps of the calculation are listed below:

(1) Multiply the images by a Tukey window to make them circularly symmetric

A frequency-domain approach allows us to estimate the horizontal and vertical shift and the (planar) rotation separately. Tukey window is also called the tapered cosine window. A Tukey window is a rectangular window with the first and last *r*/2 percent of the samples equal to parts of a cosine [27]. We multiply the low resolution images by a Tukey window to make them circularly symmetric, and thus avoid all boundary effects. Assume that we have a reference signal $f_{1}\left(x\right)$ and its shifted and rotated version $f_{2}\left(x\right)$:

$$f_{2}\left(x\right)=f_{1}\left(R\left(x+\Delta x\right)\right)$$

$$\mathrm{with}\;x=\left[\begin{array}{c} x_{1}\\ x_{2} \end{array}\right],\;\Delta x=\left[\begin{array}{c} \Delta x_{1}\\ \Delta x_{2} \end{array}\right],\;R=\left[\begin{array}{cc} \cos\emptyset & -\sin\emptyset \\ \sin\emptyset & \cos\emptyset \end{array}\right]$$

(2) Compute the Fourier transforms of all images

$$\begin{array}{cc} {Ϝ}_{2}\left(u\right) & =\iint_{x}f_{2}\left(x\right)e^{-j2\pi u^{T}x}dx\\ & =\iint_{x}f_{1}\left(R\left(x+\Delta x\right)\right)e^{-j2\pi u^{T}x}dx\\ & =e^{j2\pi u^{T}\Delta x}\iint_{x^{\prime }}f_{1}\left(Rx^{'}\right)e^{-j2\pi u^{T}x^{\prime }}dx^{\prime } \end{array}$$

where ${Ϝ}_{2}\left(u\right)$ is the Fourier transform of $f_{2}\left(x\right)$ and the coordinate transformation $x^{\prime }=x+\Delta x$. After another transformation $x^{\prime \prime }=Rx^{\prime }$, the relation between the amplitudes of the Fourier transforms can be computed as follows:

$$\begin{array}{cc} \left|{Ϝ}_{2}\left(u\right)\right| & =\left|e^{j2\pi u^{T}\Delta x}\iint_{x^{\prime }}f_{1}\left(Rx^{'}\right)e^{-j2\pi u^{T}x^{\prime }}dx^{\prime }\right|\\ & =\left|\iint_{x^{\prime }}f_{1}\left(Rx^{'}\right)e^{-j2\pi u^{T}x^{\prime }}dx^{\prime }\right|\\ & =\left|\iint_{x^{\prime }}f_{1}\left(x^{\prime \prime }\right)e^{-j2\pi u^{T}\left(R^{T}x^{\prime \prime }\right)}dx^{\prime \prime }\right|\\ & =\left|\iint_{x^{\prime \prime }}f_{1}\left(x^{\prime \prime }\right)e^{-j2\pi {\left(Ru\right)}^{T}x^{\prime \prime }}dx^{\prime \prime }\right|\\ & =\left|{Ϝ}_{1}\left(Ru\right)\right| \end{array}$$

where $\left|{Ϝ}_{2}\left(u\right)\right|$ is a rotated version of $\left|{Ϝ}_{1}\left(u\right)\right|$ over the same angle ϕ as the spatial domain rotation.

(3) Rotation estimation

The rotation angle between every low resolution windowed image and the reference low resolution windowed images is estimated.

$$h\left(\alpha \right)=\int_{\alpha -\Delta \alpha /2}^{\alpha +\Delta \alpha /2}\int_{0}^{\infty }\left|F\left(r,\theta \right)\right|drd\theta$$

where h(α) is computed as the average of the frequency values on a discrete grid with α − Δα/2 < *θ* < α + Δα/2 and ∈ρ < r < ρ. F(r,θ) is a discrete signal.

i Compute the polar coordinates (r,θ) of the image samples.
ii For every angle α, compute the average value hm(*α*) of the Fourier coefficients for which α−1<θ<α+1 and $0.1\rho <r<\rho_{max}$, where ρ is the image radius or half the image size.
iii The angles are expressed in degrees and hm(*α*) is evaluated every 0.1 degrees. A typical value used for $\rho_{max}$ is 0.6.
iv Find the maximum of the correlation between h1(α) and hm(α) between −30 and 30 degrees, which is the estimated rotation angle ϕm.
v Rotate the low resolution windowed image by −ϕm to cancel the rotation.

(4) Shift estimation

The horizontal and vertical shifts between forward and backward resampled image and the nadir image are estimated.

(a) Compute the phase difference between low resolution windowed image and the reference image.
  A shift of the image parallel to the image plane can be expressed in the Fourier domain as a linear phase shift:
  $$\begin{array}{cc} {Ϝ}_{2}\left(u\right) & =\iint_{x}f_{2}\left(x\right)e^{-j2\pi u^{T}x}dx\\ & =\iint_{x}f_{1}\left(\left(x+\Delta x\right)\right)e^{-j2\pi u^{T}x}dx\\ & =e^{j2\pi u^{T}\Delta x}\iint_{x^{\prime }}f_{1}\left(x^{\prime }\right)e^{-j2\pi u^{T}x^{\prime }}dx^{\prime }\\ & =e^{j2\pi u^{T}\Delta x}{Ϝ}_{1}\left(u\right) \end{array}$$
  It is well known that the shift parameters Δx can thus be computed as the slope of the phase difference $<\left({Ϝ}_{2}\left(u\right)/{Ϝ}_{1}\left(u\right)\right)$.
(b) For all frequencies $-u_{s}+u_{max}<u<u_{s}-u_{max}$, write the linear equation describing a plane through the computed phase difference with unknown slopes Δx.
(c) Find the shift parameters $\Delta x_{m}$ as the least-squares solution of the equations.

According to this calculation, we found the shift between different images. Then, with the shift, we registered the forward and backward images to the nadir image and made sure these three images were aligned pixel by pixel.

#### 2.2.2. Robust Super-Resolution

In [24], the study uses a median estimator to calculate the sum during the iterative framework. This is a robust method, which has a low computational cost, and is without distinguishable curacy loss. Considering the presence of outliers, this method is quite useful and valuable. The authors of [24] used the robust median-based estimator to discard the measurements, which are not consistent with the imaging model. Local model inconsistencies, which includes highlights, moving objects, parallax, can be handled at a low computation cost.

The images are reordered in column vectors in the notational framework. Via this framework, basic operations in the image formation model are linear in the image intensities. Then it can be represented as matrices operating on the vector images. Given *n* input images *g1*,…, *gn*, the image formation process of *gk* from the super-resolved image *f* can be formulated as follows:

$$\vec{Y_{k}}=D_{k}C_{k}F_{k}\vec{X}+\vec{E_{k}}$$

where $\vec{X}$ is the high-resolution image *f* reordered in a vector. $\vec{Y_{k}}$ is the *k*-th input image *gk* reordered in a vector. $\vec{E_{k}}\;$is the normally distributed additive noise reordered in a vector. $F_{k}$ is the geometric warp matrix. $C_{k}$ is the blurring matrix. $D_{k}$ is the decimation matrix.

The total squared error of the image f (represented by $\vec{X}$) is as follows:

$$L\left(\vec{X}\right)=\frac{1}{2}\overset{n}{\underset{k=1}{\sum }}||\vec{Y_{k}}-D_{k}C_{k}F_{k}\vec{X}{\left|\right|}_{2}^{2}$$

Taking the derivative of L with respect to $\vec{X}$, the gradient of L is the sum of gradients computed over the input images:

$$\vec{B_{k}}=F_{k}^{T}C_{k}^{T}D_{k}^{T}\left(D_{k}C_{k}F_{k}\vec{X}-\vec{Y_{k}}\right)$$

$$\nabla L\left(\vec{X}\right)=\sum_{k=1}^{n}\vec{B_{k}}$$

The simplest gradient-based iterative minimization method updates the solution estimate in each iteration by

$${\vec{X}}^{n+1}={\vec{X}}^{n}+\lambda \nabla L\left(\vec{X}\right)$$

where λ is a scale factor denoting the step size in the direction of the gradient.

To introduce robustness to the procedure in the equation below, the sum of images is replaced with a scaled pixel-wise median:

$$\nabla L\left(\vec{X}\right)\left(x,y\right)\approx n\cdot median{\left\{\vec{B_{k}}\left(x,y\right)\right\}}_{k}^{n}=1$$

For a symmetric distribution, a median can approximate the mean quite accurately given a sufficient set of samples. In the case of distant outliers, the median is much more robust than the mean.

#### 2.2.3. Super-Resolution Image of ZY-3 TLC

In our experiment, according to the motion estimation algorithm and robust SR processing, we aimed to achieve the SR image for ZY-3 TLC images by following the steps below:

Resample the forward and backward images. Register the nadir image and the resampled forward and backward images using the motion estimation method proposed by Vandewalle [16]. Next, reconstruct the registered nadir image and resampled forward image via the method of robust super-resolution [24]. Thus, a higher-resolution image is created from the nadir image and resampled forward and backward images. Meanwhile, similar processing is carried out for the nadir image and the resampled backward image.

**Figure 1:**
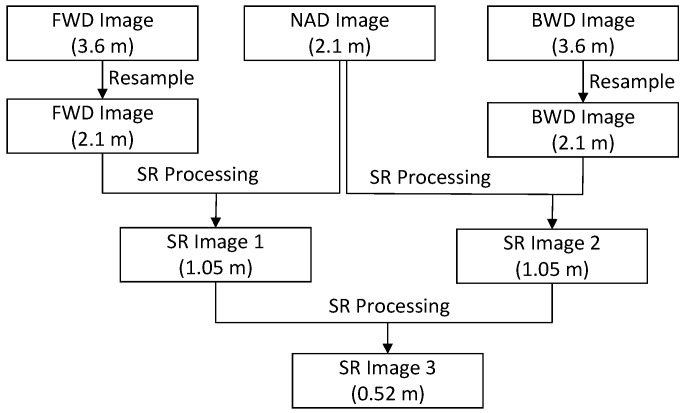
Workflow of ZY-3 Three Line Camera (TLC) images super resolution reconstruction.

At this point, two higher-resolution images with about 1.05-m resolution are created from the nadir image and forward image and the nadir image and backward image. Then, the same motion estimation method and robust SR reconstruction method are used for these two higher-resolution images. Finally, a higher-resolution image with about 0.52-m resolution is produced from the TLC images (Figure 1). The SR process is carried out in MATLAB [28] software.

## 3. Results and Discussion

### 3.1. Results

**Figure 2:**
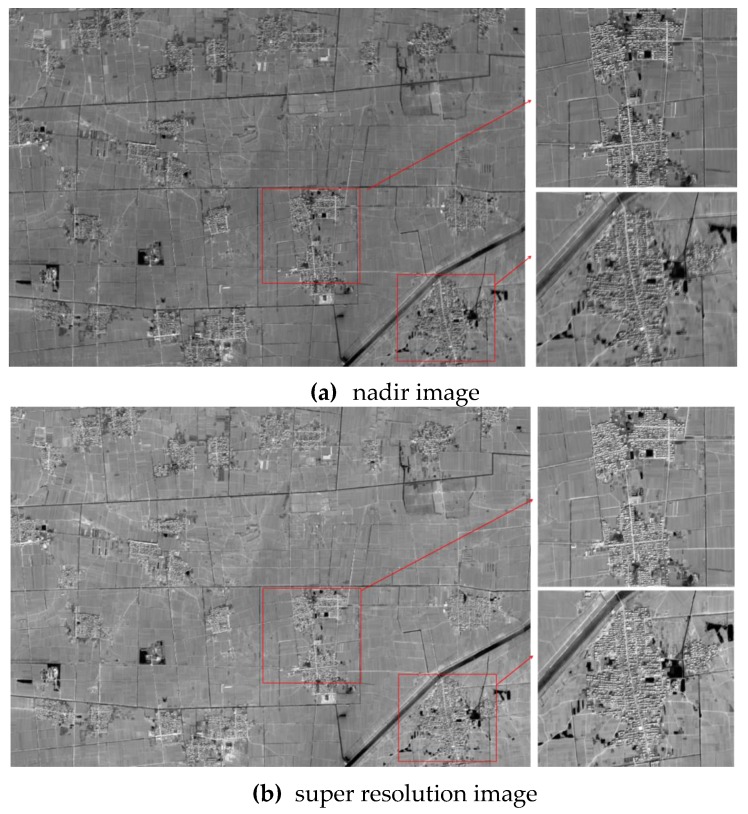
Experimental ZY-3 TLC nadir images and super resolution images. (**a**) is the original nadir image with partial enlarged views. (**b**) is the corresponding super resolution image with partial enlarged views. For better comparison, we display the nadir image and super-resolution (SR) image in the same scale.

With the help of motion estimation and robust SR processing, an SR image of ZY-3 TLC images is developed. As shown in the SR image, the clarity, sharpness, and detail are obviously improved compared with those of the original nadir image, forward image, and backward image (Figure 2).

From the image quality aspect, in this experiment, an analysis of image grayscale statistics and texture statistics of ZY-3 TLC images and the SR image was conducted. In this way, the effect of the TLC image and SR image can be assessed. The grayscale statistics included the spectral mean and spectral standard deviation, while the texture statistics included the entropy, variance, contrast, second moment, homogeneity and relative edge response [29] (Table 1).

The results of the image quality assessment reveal that the SR image has better quality with respect to most of the image quality factors. The spectral mean values of SR images are all higher than those of the original image. In addition, the standard deviation of the SR image has a higher value, especially the SR image produced from all TLC images.

**Figure 3:**
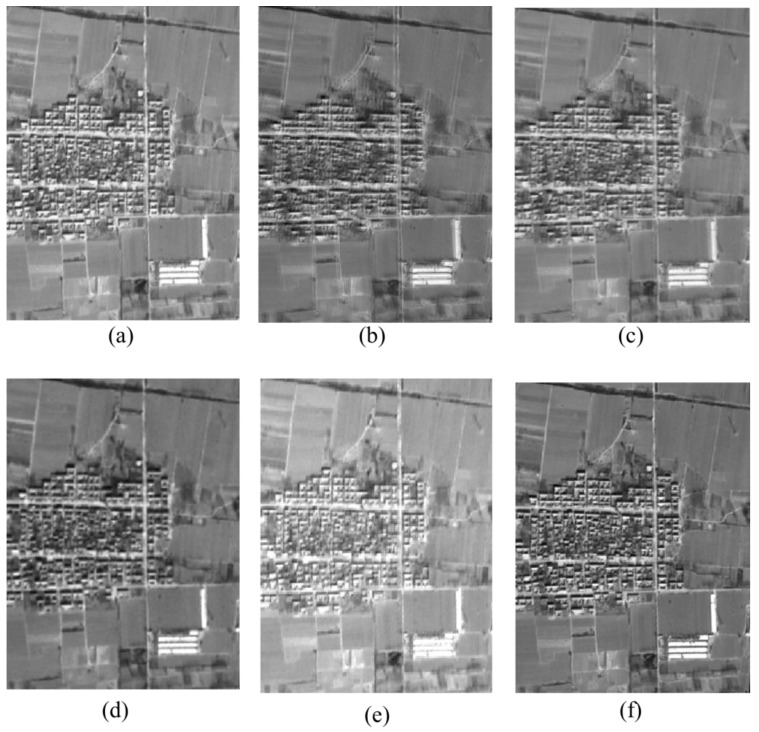
Comparison of the experimental ZY-3 image from nadir image, forward image, backward image, and super resolution image from forward image + nadir image, and super resolution image from backward image + nadir image, and SR image for all TLC images: (**a**) the nadir image; (**b**) the forward image; (**c**)the backward image; (**d**) the super resolution image from the nadir image + forward image; (**e**) the super resolution image from the nadir image + the backward image; (**f**) the super resolution from (**d**) + (**e**).

**Figure 4:**
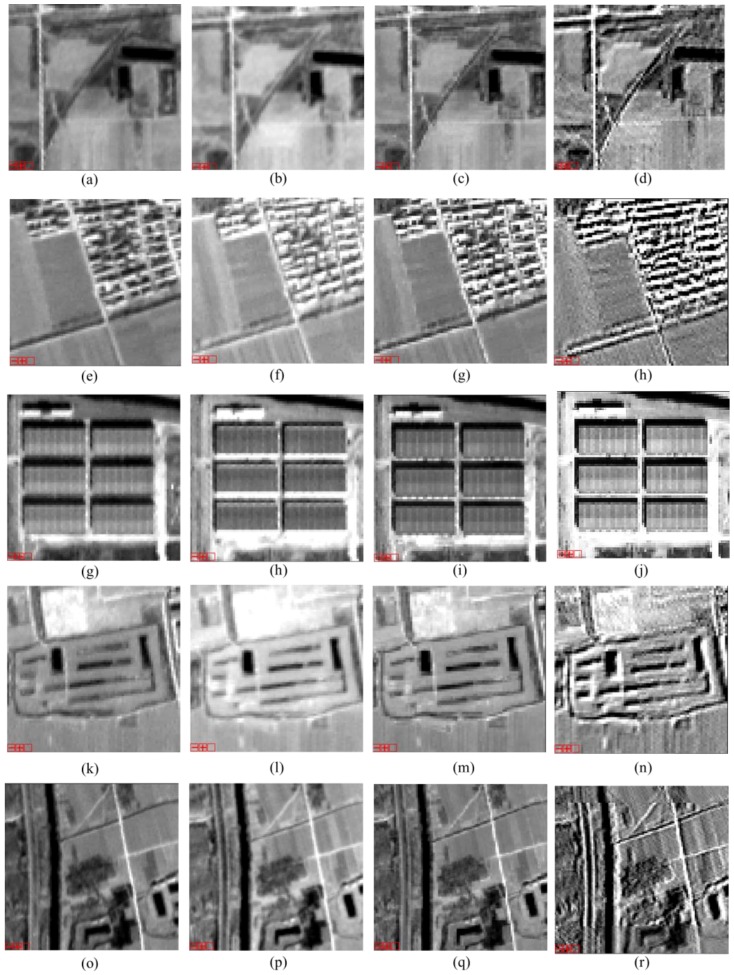
Detail comparisons of experimental ZY-3 images and corresponding super resolution images for different ground objects. These images are all clipped from our experimental images. From left to right of each column, the images are the forward images, the backward images, the nadir images, and their super resolution images, respectively. Different rows show different ground objects, and images from to the up down describe the paths and the canals, the paths and resident buildings, the factory buildings, the reservoirs and lands, the paths and lands, respectively . For example, from (**a**–**d**), we can see clearly the path and the canal in super resolution image (**d**), which are too blur to be identified in each individual ZY-3 image (the forward image (**a**), the backward image (**b**), and the nadir image (**c**)).

From the image texture aspect, the SR image has better texture performance. Entropy, variance, contrast, and second moment results are all higher for the SR image, showing that the texture of the SR image is richer and finer. The homogeneity of the SR image is comparatively low, i.e., it has greater variance for its detail. A comparison of the experimental images from different sources is shown in Figure 3, and some details are shown in Figure 3 and Figure 4, which provide additional examples of the experimental images.

### 3.2. Discussion

In our experiment, higher resolution and better image quality were achieved in the SR image derived from the ZY-3 TLC images. With the help of SR reconstruction, the combined ZY-3 TLC images provided a higher-resolution image for the same area. This finding has great value for the study of image processing for ZY-3 and similar satellite data.

Our experiment shows that using the motion estimation method of Vandewalle et al., which computes images’ Fourier transforms and determines the 1-D shifts in both their amplitudes and phases, has the advantage of discarding high-frequency components, where aliasing may have occurred, to be more robust. This method is suitable for the ZY-3 TLC images. Although the TLC images are acquired from different angles, including forward angles, backward angles, and nadir, their acquisition times can be considered the same. Some shift and rotation exists in the TLC images. Via the motion estimation method used here, we can register the TLC images correctly.

The robust SR method, which uses a median-based estimator to discard measurements that are inconsistent with the imaging model, is proven capable of handling local model inconsistencies, such as different highlights and the moving effect of TLC images. From the experimental results, the clearer and sharper edge shows that the image quality is improved significantly, though some of the textures may appear as noisy points. Overall, SR reconstruction improves the resolution of ZY-3 TLC images. According to the calculation, the SR image resolution should be half that of the original nadir image.

However, because we resampled the forward image and backward images to a higher resolution, the resolution of SR results of forward image and nadir image, backward image and nadir image are not actually equal to half of the nadir image, nor is the resolution of the final SR results. In other words, although the final SR image resolution is 0.52 m, the quality of this image is not as high as that of the actual 0.52 m resolution image. We could consider that the 0.52 m final resolution in our experiment is only a calculated value, not the actual resolution. The image is estimated to be approximately 1 m resolution in its image quality and performance. The exact resolution of the final SR image is worthy of further research. However, it is clear that after SR processing of ZY-3 TLC images, a much higher-resolution image is made available.

## 4. Conclusions

In this study, super-resolution reconstruction of ZY-3 TLC images is conducted. The motion estimation method, which computes images’ Fourier transforms and determines the 1-D shifts in both their amplitude and phase, is used to register the TLC images. Then, the robust super-resolution reconstruction for the recorded images is conducted to achieve the final SR image. The results show good quality in both the image grayscale characteristic and texture effect; moreover, significantly higher resolution can be proved for our SR processing.

With the help of super-resolution reconstruction of ZY-3 TLC images, the limitation of ZY-3 panchromatic resolution in image analysis, image extraction, and image mapping is reduced; specifically, this technique may be useful for applications that require resolution higher than 2 m. With this approach, ZY-3 images can achieve higher resolution and be used in a wider range of applications. Based on the SR method of this study, we would carry out more experiments for ZY-3 TLC images in the future. For example, we would use the ZY-3 TLC image and SR method to extract a built-up area. In other words, this study is just the beginning of our ZY-3 TLC SR image application.

## Figures and Tables

**Table 1:** Comparison of the image quality. FWD is the forward image; BWD is the backward image, NAD is the nadir image; F + N is the SR image of forward image and nadir image; B + N is the SR image of backward image and nadir image; F + B + N is the final SR image from F + N and B + N.

	Mean	Standard Deviation	Entropy	Variance	Contrast	Second Moment	Homogeneity	Relative Edge Response
FWD	112.90	36.79	7.09	7.83	17.22	0.22	0.59	0.17
BWD	130.34	42.85	6.98	8.15	18.00	0.27	0.54	0.18
NAD	120.51	38.74	7.11	11.69	27.72	0.31	0.49	0.20
F + N	122.45	39.71	7.09	9.09	28.19	0.24	0.48	0.18
B + N	129.12	43.37	7.13	5.08	11.77	0.31	0.62	0.20
F + B + N	131.12	47.67	7.14	13.32	35.23	0.32	0.44	0.22
